# Understanding Engagement in HIV Programmes: How Health Services Can Adapt to Ensure No One Is Left Behind

**DOI:** 10.1007/s11904-020-00522-1

**Published:** 2020-08-26

**Authors:** Anna Grimsrud, Lynne Wilkinson, Ingrid Eshun-Wilson, Charles Holmes, Izukanji Sikazwe, Ingrid T. Katz

**Affiliations:** 1 International AIDS Society, 3 Doris Road, Claremont, Cape Town, 7708 South Africa; 2 Desmond Tutu HIV Centre, University of Cape Town, Anzio Road, Observatory, Cape Town, 7925 South Africa; 3 Department of Public Health Medicine, School of Public Health and Family Medicine, University of Cape Town, Cape Town, South Africa; 4 School of Medicine, Washington University, St Louis, MO USA; 5 Center for Innovation in Global Health, Georgetown University, Washington, DC USA; 6 Centre for Infectious Disease Research in Zambia, Lusaka, Zambia; 7 Department of Medicine, Brigham and Women’s Hospital, Boston, MA USA; 8 Harvard Medical School, Boston, MA USA; 9 Massachusetts General Hospital Center for Global Health, Boston, MA USA; 10 Harvard Global Health Institute, Cambridge, MA USA

**Keywords:** Engagement, Re-engagement, Retention, HIV, Client-centred, Differentiated service delivery

## Abstract

**Purpose of Review:**

Despite the significant progress in the HIV response, gaps remain in ensuring engagement in care to support life-long medication adherence and viral suppression. This review sought to describe the different points in the HIV care cascade where people living with HIV were not engaging and highlight promising interventions.

**Recent Findings:**

There are opportunities to improve engagement both between testing and treatment and to support re-engagement in care for those in a treatment interruption. The gap between testing and treatment includes people who know their HIV status and people who do not know their status. People in a treatment interruption include those who interrupt immediately following initiation, early on in their treatment (first 6 months) and late (after 6 months or more on ART). For each of these groups, specific interventions are required to support improved engagement.

**Summary:**

There are diverse needs and specific populations of people living with HIV who are not engaged in care, and differentiated service delivery interventions are required to meet their needs and expectations. For the HIV response to realise the 2030 targets, engagement will need to be supported by quality care and patient choice combined with empowered patients who are treatment literate and have been supported to improve self-management.

## Introduction

Set in 2014, the UNAIDS Fast-Track targets for accelerating the HIV response to reach the 90-90-90 goals of 90% of people knowing their status, 90% of those being on treatment and 90% of those on treatment being virally suppressed are due at the end of this year (2020) [1]. At the time these goals were set, less than 40% (13.6 million) of the 25 million people living with HIV were accessing antiretroviral therapy. By the end of 2018, global estimates were 79-78-86, highlighting the significant progress that has been made [2]. However, gaps remain and health services need to adapt to ensure that no one is left behind.

As we get closer to the targets, it is more challenging to reach those who are not yet diagnosed or who are not engaged in care. While there have been significant efforts to expand HIV treatment programmes through efficiencies and effectiveness, we now need to acknowledge that we may have achieved most of the “easy wins” and it is going to take something extra to reach the last mile.

There are opportunities to improve healthcare systems to both more fully meet the needs of individuals who are not engaged in care and do more to enable people in HIV care towards improved self-management. Critical to this process of improvement is the need to ensure opportunities for shared decision-making and mutual respect between people living with HIV and the healthcare system for HIV care—all while acknowledging that many reasons for missing care are unintentional [3]. Shifting away from stigmatising language with labels including “hard to reach”, “defaulter” and “treatment refuser” is an important part of this evolution towards more person-centred care.

The current HIV response is in transition. Today nearly four out of every five people living with HIV (79%) knows their status [4]. Despite the recommendation to treat all people living with HIV, it is estimated that a ***“***worryingly large proportion of people diagnosed with HIV—more than 20%—had not yet initiated treatment in 2018” [4]. To grapple with this challenge, we need to go further than *knowing* one’s status to support *accepting* and *understanding*. And this gap—the one between the first and second 90—is only one of the places in the HIV care cascade where we see challenges in engagement.

Addressing this gap requires programme innovation and high-quality implementation research [5, 6], to answer how best to provide HIV care. What works? Where? And for whom? We need “the right research at the right time in the right context” [7]. This ongoing transition to implementation research is accompanied by a trend towards differentiated service delivery, an approach that is “client-centred” and acknowledges client choice in how they receive treatment, care and support [8, 9]. It is implementation research that stands to answer some of these pressing questions regarding engagement in HIV programmes.

We will define the different points in the HIV care cascade where people living with HIV may become disengaged, review the literature for each and highlight promising interventions that can be considered to address these challenges, improve engagement and reach that last mile.

## Different Groups of Patients Not Engaged in Care

**Fig. 1:**
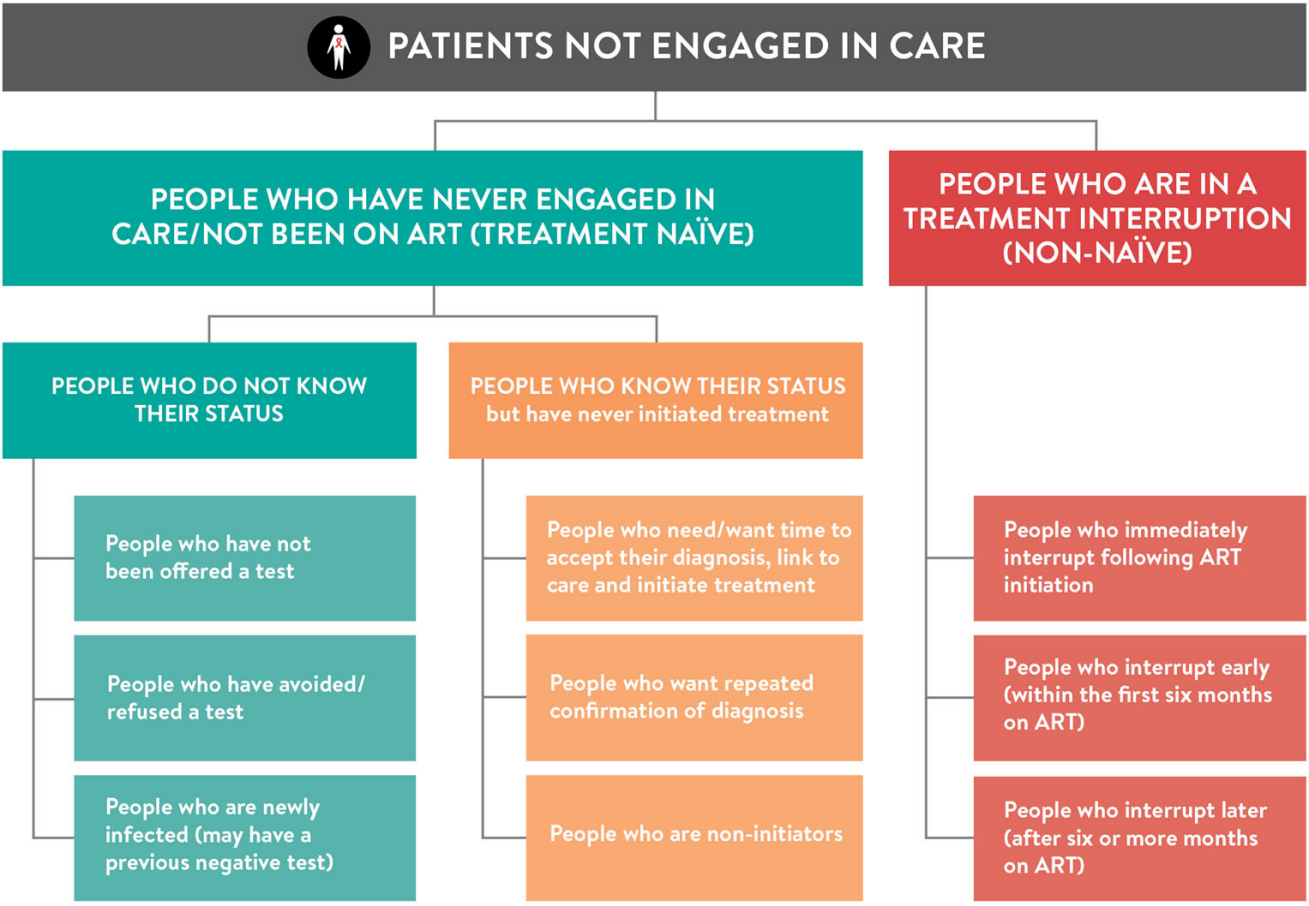
Schematic depiction of people living with HIV who are not in care

Identifying the different groups of non-engaged individuals across the cascade (Fig. 1) can help ensure which interventions are needed to support better engagement in care among people living with HIV and where there may be gaps. Critically, these are not mutually exclusive groups, and the same individual can be in more than one group, and in some instances, more than one concurrently, during the life course of HIV. While previous models of behaviour change interventions, including the Capability-Opportunity-Motivation framework from Michie et al. [10], have been used to ground behaviour change interventions, Fig. 1 moves beyond the individual and their capacity, to health systems interventions that better meet the needs and expectations of people who are not engaged in care.

There are two distinct categories of people who are not engaged in care that need to be considered: (1) people who have never engaged in care and have therefore not been on ART (treatment naïve) and (2) people who are in a treatment interruption (non-naïve). Each of these groups can be broken down further.

### People Who Have Never Engaged in Care/Not Been on ART (Treatment Naïve)

Among those never engaged in care will be (a) people who do not know their status and (b) people who know their status and have never initiated treatment. The gap in those who are unaware of their status has steadily decreased over time as HIV testing has increased. Data from the nationally representative Population-based HIV Impact Assessment (PHIA) surveys reveal consistent trends of lower knowledge of status of young adults, adolescents and men compared with women [11]. A wealth of evidence also highlights gaps in knowledge of HIV status among key populations [12].

#### People Living with HIV Who Do Not Know Their Status

This is a first 90 gap and is comprised of (i) people not offered a test, (ii) people avoiding or refusing to test and (iii) people who are newly infected and may have tested negative on previous HIV tests (Fig. 1). Recent data from Malawi provide a rebuttal to the often-cited falsehood of “poor men’s health seeking behaviour”. In a cross-sectional survey in two areas of Malawi, 94% of men had visited a health facility in the past 2 years—79% as a patient and 81% as a guardian. However, only 24% of clients and 12% of guardians were offered an HIV test [13]. In the Kwa-Zulu Natal province of South Africa, refusal rates for HIV testing decreased from 70 in 2008 to 41% in 2016, but only 20% of the population consistently consented to testing when offered annually [14•]. Finally, those who are newly infected require timely and possibly frequent testing to support early diagnoses and timely engagement in care. Data from the Evidence for Contraceptive Options and HIV Outcomes (ECHO) trial highlighted the continued high rates of incident infection among younger women, with those under 25 years of age having higher rates of seroconversion compared with women 25 to 35 years of age [15].

#### People Who Know Their Status but Have Never Initiated Treatment

In addition to the evidence of treatment as prevention from the HPTN052 study [16], part of the rationale for “test and treat” or “treat all” was to reduce losses in the pre-ART phase [17, 18]. Despite widespread global uptake of “treat all” into guidelines, however, there is still a persistent and considerable group of people who receive an HIV-positive diagnosis and do not initiate treatment [19]. In the HPTN 071 (PopART) study, approximately 30% of people who tested positive had not initiated treatment 12 months later, despite linkage support strategies [20•]. Patients in this group include people who need or want more time to accept their diagnosis before treatment initiation, those who want repeated confirmation of their diagnosis and those who are non-initiators (previously referred to as “treatment refusers”) and face challenges in engaging in care [21, 22] (Fig. 1).

With the current emphasis and targets for same-day ART initiation, Seeley et al. make the case that for some people, particularly those who are asymptomatic at diagnosis, “a brief ‘pre-ART’ period may serve as an opportunity to come to terms with [their] HIV status prior to commencing ART” [20•]. Reduced readiness to start ART has been strongly associated with poorer linkage [23] and associated with not expecting to test HIV positive, whereas higher readiness has been associated with better ART knowledge and knowing someone who has experienced the positive effects of treatment [24]. The CASCADE trial in Lesotho provides further evidence that some patients may require more time before starting treatment. While patients in the same-day arm had better early outcomes, after 24 months, there was no difference between the same-day and standard of care arms in regard to suppression and retention at 24 months [25].

There is also a small, but critical amount of data emerging on the frequency of repeat testing among those with an HIV diagnosis. In the Western Cape province of South Africa, of all those presenting for HIV testing at a health facility, 16% had a previous HIV positive diagnosis and among those who were positive, 75% had been previously diagnosed [26]. While there is provider resistance, often driven by funder priorities [27], for repeat HIV testing among those previously diagnosed, linkage and ART initiation or re-engagement appear to increase among the retesting group. Recent modelling of the South African epidemic in regard to the impact of future HIV testing strategies highlights that “much of the modelled benefit of testing arises from retesting previously-diagnosed individuals who have either never linked to HIV care or dropped out of care” [28••]. Data from Uganda also reported high volumes of retesting—over a third (37%) of those testing positive already knew their status. The odds of retesting were higher in women compared with men and in those with more years of education [29]. In Ethiopia, patients were interviewed after initiating ART and 13% reported repeat testing. Repeat testing was associated with having doubted their HIV status and initiating treatment at a different facility from where they tested and was less likely among those who had tested for HIV because of symptoms [30].

Among those who are non-initiators, the CASCADE trial found that despite consenting to home-based testing before randomisation to the different intervention arms, 13% (36/274) of people who were HIV-positive did not seek HIV care over the 2 years of the study; “the majority rejected contact with the health system or were unwilling to take ART” [25].

### People Who Are in a Treatment Interruption

As the number of people ever initiated on ART continues to grow, there is an increasing pool of people who will potentially interrupt treatment. Those who are in a treatment interruption (not ART naïve) include (a) immediate interrupters (those who do not return after ART initiation), (b) early interrupters (those who interrupt in the first six months on ART) and (c) those who interrupt later, after 6 or more months on ART (Fig. 1). This differentiation is important for discussing different targeted interventions.

This first group—(a) immediate interrupters—is likely similar to those who were not ready and needed time to accept their diagnosis before initiating treatment but may have started ART because of the emphasis on same-day and rapid start [31•]. In analyses from two districts in South Africa, those who initiated treatment on the same-day had a larger initial drop in immediate loss (within the first month) compared with those who took longer to initiate treatment. More than a third (35%) of same day initiators did not return after the initiation visit.

Analyses from the Centre for Infectious Disease Research in Zambia (CIDRZ) group explored engagement trajectories [32••] and treatment outcomes in the second two groups (b) and (c) of early and late interrupters through sampling those disengaged from care [33, 34]*.* In the work on treatment trajectories, six groups of patients are described including three groups that disengage or are LTFU—early LTFU with late re-engagement, early LTFU without re-engagement and late LTFU without reengagement. All three of these groups had higher rates of mortality compared with those with consistent adherence and retention, consistent with other data that 71% of those who disengage have high viremia when traced [34•].

Temporal trends highlight that an increasing proportion of people living with HIV are ART experienced. In the Western Cape, data from 2008 to 2017 highlights a dramatic increase in the number of people accessing ART and ART coverage, with a concurrent decrease in the proportion of people initiating with AHD (advanced HIV disease: defined as a CD4 cell count below 50 copies/mL). Over the same time period, the proportion of people with AHD presenting as treatment experienced increased from 14% to 57%, and in 2016, 51.8% were ART experienced, of whom 76% could be confirmed to be off ART or had recent viremia” [35•]. Similarly, in data from Kenya, Malawi and South Africa, 10% of all people living with HIV had AHD of which 63% were aware of their status, but only 40% were currently on ART [36•]. These data emphasise that going forward, we need to ensure that the health system is ready to support patients re-engaging in care, while concurrently reducing the likelihood of disengagement. Other data from South Africa highlights that linkage to care within three months of testing positive was similar for those who had been previously diagnosed but never engaged (adjusted odds ratio (aOR): 0.97) and substantially higher in those previously in care but lost to follow-up (LTFU) (LTFU > 24 months aOR: 1.44, LTFU 13–24 months aOR: 2.52) compared with those newly diagnosed and never in care [37••]*.*

## Beyond Treatment Naïve and Non-naive

In addition to describing those not engaged or not in care as treatment naïve or non-naïve, the groups can be divided into those who are “well” and those with AHD. For the purposes of differentiating services, it is important to consider the clinical characteristics, specific population(s) (age, gender, key population, pregnant and breastfeeding women, etc.) and the context of those not-engaged. These three axes have previously been described as the “elements” of differentiated service delivery [38].

The clinical differentiation between people who are well and those with AHD is particularly vital to ensure timely and appropriate access to diagnostics and treatment of the large proportion of those disengaged who have AHD. This package of care for AHD is well defined within the 2017 World Health Organization guidelines, which outline the different clinical packages of HIV care for those who are initiating or reinitiating and are well and those who are initiating or reinitiating and have AHD [39]. It is important to highlight that many programmes currently risk being seriously compromised if they do not have access to or have deprioritised baseline CD4—required to define AHD status [40].

## Where to from Here: the Path Forward

### Interventions to Support Improved Engagement

With the successful scale-up of HIV testing and treatment, the challenges that remain to ensure no one is left behind require a shift in the HIV response towards supporting adherence and retention and going beyond knowledge of one’s HIV status to acceptance and understanding. Further, it is about understanding people’s preferences and barriers and working towards supporting more diverse pathways to long-term retention. This remains more challenging since health systems are not set up to “welcome back” non-naïve clients and much of the early emphasis on treatment education and peer providers to support ART initiation has fallen away. While HIV programmes have differentiated services to support long-term adherence by simplifying and spacing treatment collection with options closer to home, patients’ awareness of these options remains limited prior to eligibility assessment. Further, to understand the nuances of engaging patients requires a fundamental shift away from framing the HIV care cascade as linear and acknowledging that it is in fact much more circular [41] with patients continually evaluating their decision to engage and remain engaged in care [42].

Using the categories from Fig. 1, Table 1 outlines some of the possible strategies to support improved engagement at different levels. These interventions may apply to multiple groups of patients, given that as noted, the patient groups described are not mutually exclusive.

**Table 1:** Strategies to improve engagement in HIV care

Intervention target level	People who have never engaged in care/not been on ART (treatment naïve)		People who are in a treatment interruption
	People who do not know their status	People who know their status	
Individual	• HIV self-testing	• Strategies to facilitate acceptance of HIV status including repeated retesting	• Improved “adherence” counselling which addresses broader psychosocial challenges
		• Increase awareness of treatment journey ahead and access to differentiated service delivery options	• Increase awareness of treatment journey ahead and access to differentiated service delivery options
		• Offer of rapid ART initiation	
		• Improved post-test counselling that addresses “real-life concerns” and ART fears	
Interpersonal	• Secondary distribution HIVST	• Disclosure assistance	• Improved “adherence” counselling which addresses broader psychosocial challenges
	• Social network HIV testing approaches	• Couples testing	
	• Couples testing	• Peer support interventions	• Peer support interventions
		• Male partner participation in HIV care for women	
		• Family-centred care	
Health system and other organisations	• Improved quality HIV testing patient experience	• Improved quality HIV testing patient experience	• Facilitation of re-engagement in care (“welcome back”) and transfers between clinics including friendly provider attitudes
	• Workplace HIV testing services	• Facilitation of repeat HIV testing to confirm diagnosis	
	• Increase PITC including for those accompanying others to facilities	• Strategies to improve linkage after testing and accommodate delays	• Tracing interventions
			• Improved service quality to provide more “patient-centred care” at all healthcare levels (fast-track services, adherence clubs, multi-month scripting, adolescent friendly services, etc.)
	• Offer repeat HIV testing, particularly for key-populations		
Community	• Community-based HIV testing and treatment strategies	• Community-based ART initiation	• Community-based differentiated service delivery as a component of patient-centred care
	• Strategies to increase community awareness of U=U	• Strategies to increase community awareness of U=U	• Strategies to destigmatise HIV in communities
	• Strategies to increase awareness of treatment journey ahead and access to differentiated service delivery options	• Strategies to destigmatise HIV in communities	
Policy	• Increasing DSD enabling policies (as above) • Decriminalisation of key populations and advocacy for human rights		

There are some data on interventions that could support engagement in the HIV care cascade. For those never in care who do not know their status, it is critical to reach them with testing approaches that work for them. At a minimum, offering testing options when they do interact with the health system or when their family or social network interact with the health system while recognising that specific populations are unlikely to be reached without targeted community testing [43, 44]. HIV testing should continue to be routinely offered at all entry points at health facilities in high prevalence areas through optimising provider-initiated counselling and testing [45•]. We also need to challenge assumptions that may be outdated or incorrect—including that men have poor health seeking behaviour or do not attend health facilities. As noted above, recent data from Malawi highlighted that 92% of men had accessed a clinic in the past 2 years, but the vast majority were not offered testing for HIV [13].

HIV self-testing has an important role for men, younger people and key populations with lower testing rates. High uptake of HIV self-testing (HIVST) has been observed through direct distribution at facilities [45•], secondary distribution to partners of antenatal clients [46] and community distribution to younger male, [47] and key populations outside of health facilities [48, 49]. If coupled with effective linkage strategies, self-testing could have a substantial impact on cascade targets in these population sub-groups [50]. For those newly infected, it is critical that HIV retesting is funded and offered at regular intervals, particularly in populations that are especially vulnerable such as adolescent girls and young women, key populations and migrants [33].

In addition to reconsidering testing approaches, out-of-facility or community-based ART initiation may also increase engagement in care. A growing body of evidence from Lesotho [51], South Africa [52] and Tanzania [53] highlights high levels of engagement with community-based ART initiation among populations previously not engaged in care.

### Interventions to Support Those Who Know Their Status but Are Not Engaged in Care

Among those who have received an HIV diagnosis but not engaged in care, the interventions need to focus on acceptance of HIV status and understanding to increase ART uptake. While evidence on how to do this is limited, some qualitative work highlights that the testing experience itself influences future engagement in care [54]. The messaging received during the testing process is also critical, and discussions that do not address “future care-seeking concerns” present challenges to long-term engagement in care. Interventions for those who want or need longer to link include reducing healthcare worker stigma towards these individuals and a balancing of treatment literacy, peer engagement and counselling to support readiness and linkage without either obliging people to start immediately. For those who need confirmation of diagnosis, modelling work from South Africa provides data on the utility of retesting and the need to reconsider whether retesting is indeed a wasted intervention. As with those who are resistant to testing, those who have a diagnosis and are resistant to treatment require interventions that reframe “HIV and ART from ‘losing’ to winning’ alongside services that are “more convenient, responsive and empowering for *all* patients” [55].

Further, the value of the first 90 is waning the closer we come to reaching it. Successful testing should not be defined simply by “knowing one’s status”, but by acting upon this knowledge and engaging in care. As such, testing programmes require capacity to offer this additional engagement. The importance placed on testing targets need to shift and reflect that testing without additional engagement is not helpful.

### Interventions to Support Re-engagement Among Patients in a Treatment Interruption

For those in the midst of a treatment interruption, it is important to both reduce the likelihood of these interruptions occurring and ensure timeous return to care [56•]. While challenging to ensure, the importance of provider attitude and friendliness cannot be unscored. Empiric work from Zambia and Mozambique suggests that health services should be welcoming or, at a minimum, not viewed as punishing and uninviting [57, 58]. Health services need to acknowledge that a growing number of HIV patients are returning to care and be prepared to welcome them back and support addressing barriers that were associated with their disengagement—such as frequent clinic visits, long waiting times or poor understanding of Undetectable = Untransmittable (U=U) [59••].

To address those who are immediately disengaged from care following ART initiation, we need to prioritise a *quality* ART initiation experience over just same-day ART start. There is substantial early loss after treatment initiation that largely goes unacknowledged. In Zambia, more than a third of all patients (36%) lost to follow-up between October 2018 and September 2019 had initiated treatment in the past year [60]. Data from South Africa highlights that in 2019, 23% of those initiated had disengaged by 3 months, 30% by 6 months and 36% by 12 months after initiation [61]. There needs to be real choice regarding timing of ART initiation and sufficient and appropriate treatment education provided in the first and subsequent visits [62] to support patient empowerment and understanding.

The subsequent visits must include quality counselling and provide patients with details of the pathway ahead as they seek to “return to normal life” following their HIV diagnosis. Further, with the advent of dolutegravir offering quicker viral suppression and the scale-up of differentiated ART delivery models, eligibility for differentiated ART delivery after one suppressed viral load should be considered to support patient-centred care. Patients may also benefit from increased knowledge of and access to differentiated ART delivery models—including group models such as Adherence Clubs [63] and community ART groups [64, 65] and community-based individual models including collection from private pharmacies [66] and through community drug distribution points [67].

For those with advanced HIV disease, engagement in care is of course critical to morbidity and mortality outcomes. From initiation, closer case management is required including visits from community health workers, being prioritised for tracing following missed visits and facilitated linkage to care. Beyond six months, engagement will be supported by quality care and patient choice [68•] for service delivery combined with empowered patients that are treatment literate as well as recognising that patient needs change over a lifetime [57•].

## Conclusions

The HIV response has successfully improved access and uptake of HIV testing and treatment services. To see sustained gains towards the 2030 UNAIDS targets, it will be critical to improve engagement among all people living with HIV—both those who have never been engaged in care and those in a treatment interruption. HIV programmes need to adapt to the specific needs of an increasing proportion of people initiating treatment who are non-naïve and re-engaging in care. People living with HIV need to be at the core of the response, empowered to self-manage and be supported by a health system that acknowledges the challenges of life-long chronic disease management.
